# Whole-Genome Sequence of Streptococcus pyogenes Strain 591, Belonging to the Genotype *emm*49

**DOI:** 10.1128/MRA.00816-21

**Published:** 2021-10-28

**Authors:** Nadja Patenge, Christian Rückert, Jana Bull, Kevin Strey, Joern Kalinowski, Bernd Kreikemeyer

**Affiliations:** a Institute of Medical Microbiology, Virology and Hygiene, University Medicine Rostock, Rostock, Germany; b Microbial Genomics and Biotechnology, Center for Biotechnology, Universität Bielefeld, Bielefeld, Germany; University of Rochester School of Medicine and Dentistry

## Abstract

Streptococcus pyogenes strain 591 is a clinical isolate belonging to the genotype *emm*49. It has been intensively studied for its pathogenicity traits. In this study, the complete genome of strain 591 was sequenced. It consists of a chromosome of 1,762,765 bp with a G+C content of 38.5%.

## ANNOUNCEMENT

Streptococcus pyogenes (group A Streptococcus [GAS]) is a Gram-positive, exclusively human pathogen responsible for a variety of diseases. Manifestations range from self-limiting superficial infections of the skin or throat to life-threatening invasive diseases. S. pyogenes causes up to 18 million invasive infections per year and 500,000 deaths (1). The number of invasive diseases is rising, and untreated superficial infections notoriously lead to the development of severe invasive infections or autoimmune sequelae (2, 3). S. pyogenes strain 591 is a patient skin isolate which was kindly provided by R. Lütticken (Aachen, Germany) (4). The strain belongs to the genotype *emm*49, and the molecular basis of its pathogenicity has been extensively characterized. For instance, integrin-dependent invasion into host cells and the role of FCT-3 pilus region-encoded proteins in streptococcal virulence have been studied in this strain (5, 6). Furthermore, it is one of the model strains that have been employed for the identification and functional characterization of small noncoding RNAs (ncRNAs) in S. pyogenes (7–9). To facilitate comparative genomic and transcriptomic analyses, the genome of S. pyogenes strain 591 was sequenced.

S. pyogenes strain 591 was grown overnight in Todd-Hewitt medium supplemented with 0.2% yeast (THY), and DNA was extracted using the DNeasy blood and tissue kit (Qiagen GmbH, Hilden, Germany). Libraries were prepared using the Nextera XT kit (Illumina, Inc., San Diego, CA), and initial sequencing was conducted using an Illumina MiSeq sequencer (2 × 300-nucleotide [nt] paired-end run). The read quality was checked using FastQC v0.11.3 (10) (option: -t 16). No error correction or adapter trimming were performed other than screening against the phiX library and removing 3′ stretches of Ns. The initial assembly of 1,115,170 reads (coverage, 94.3×) using Newbler v2.8 (11) (options: -large, -siom 16, -m, –consed) resulted in 61 contigs in 34 scaffolds. The *N*_50_ value of the contigs was 62,599 bp. To obtain a complete genome sequence, an additional library for Oxford Nanopore Technologies (ONT) sequencing was prepared using a rapid barcoding kit (SQK-RBK004; Oxford Nanopore Technologies, Oxford, UK) according to the manufacturer’s instructions. Sequencing was performed using a R9.5 flow cell on a MinION sequencing platform according to the manufacturer’s instructions. Base calling and demultiplexing were performed using Albacore v2.3.1. The obtained ONT data (13,578 reads; coverage, 20.3×) were assembled using Canu v1.6 (12) with the parameter genomeSize = 2m. The read *N*_50_ value for the ONT data was 9,505 nt. The reads were filtered for size, excluding reads shorter than 1 kb. The read quality was checked using FastQC. Error correction and trimming were performed as part of the Canu assembly. The resulting 8 contigs were polished with the Illumina data using Pilon v1.22 (13). Minimap2 v2.17 (parameters: -ax sr, –secondary = no), BWA-MEM v2 (14) (parameters: -O1, -E1), and Bowtie2 v2.3.2 (15) (parameters: -X 750, –no-unal) were used for mapping. Both assemblies were then merged manually using Consed (16). Circularization was performed manually in Consed using the data from the two assemblies. Rotation of the genome was performed using SnapGene (Insightful Science). The resulting gapless genome sequence consisted of 1,762,765 bp, with a G+C content of 38.5%. The final coverage, based on the assembled data, was 102.7×. Automatic annotation using Prokka v1.14.5 (17), followed by manual curation of the Rfam hits, resulted in the assignment of 1,575 genes, out of 1,640 protein-coding sequences, 67 transfer-messenger RNAs (tmRNAs), 18 rRNAs, and 31 noncoding RNAs (ncRNAs). Furthermore, 10 regulatory regions were detected using this approach. These comprised 5 riboswitches, 3 attenuators, and 2 response elements.

### Data availability.

The complete genome sequence of S. pyogenes strain 591 has been deposited in NCBI GenBank and the raw data into the NCBI Sequence Read Archive (SRA). The genome versions described in this paper are the first versions. The accession numbers are PRJNA13286 (BioProject), NZ_CP077685.1 (GenBank), SRR14857120 (SRA; Illumina data), and SRR14857119 (SRA; ONT data).
